# Personalised approach to hypertension treatment: protocol for the HYPERMARKER randomised controlled trial

**DOI:** 10.1136/bmjopen-2026-117869

**Published:** 2026-07-16

**Authors:** Matthew Chapman, Jonathan E Knikman, Alastair Mobley, Bart Lagerwaard, Fernando Martinez-Garcia, Seraphine Zeitouny, Daniel Engler, Renate B Schnabel, Wilko Spiering, Iris D Bos, A Champsi, Alexander W Carter, Thomas Hankemeier, Diederick Grobbee, Dipak Kotecha, HYPERMARKER Consortium, A Acharjee

**Affiliations:** 1 Department of Cardiovascular Sciences, University of Birmingham, Birmingham, England, UK; 2 NIHR Birmingham Biomedical Research Centre, University Hospitals Birmingham NHS Foundation Trust, Birmingham, UK; 3 Julius Center for Health Sciences and Primary Care, University Medical Centre Utrecht, Utrecht, The Netherlands; 4 Internal Medicine Department, Hypertension Unit, Clinical Hospital of Valencia, Valencia, Spain; 5 Cardiometabolic Research Group, Research Institute of the Clinical Hospital of Valencia (INCLIVA), Valencia, Spain; 6 University of Valencia, Valencia, Spain; 7 London School of Economics and Political Science, London, UK; 8 Department of Cardiology, University Heart and Vascular Center Hamburg, University Medical Center Hamburg-Eppendorf, Hamburg, Germany; 9 Department of Vascular Medicine, University Medical Centre Utrecht, Utrecht University, Utrecht, The Netherlands; 10 Metabolomics and Analytics Centre, Leiden Academic Centre for Drug Research, Leiden University, Leiden, The Netherlands

**Keywords:** Hypertension, Machine Learning, Clinical Trial, Randomized Controlled Trial, Cardiovascular Disease

## Abstract

**Introduction:**

Blood pressure treatment response is variable in individual patients, and the choice of medical therapy is often dependent on clinician experience. Treatment choices could be personalised by patient empowerment and metabolomic profiles, and augmented by machine learning, but robust evaluation is lacking on how these can be combined to enhance clinical effectiveness. The HYPERMARKER trial will evaluate how an individualised choice of medication class can address the avoidable global health and economic burdens of hypertension.

**Methods and analysis:**

The HYPERMARKER trial is a proof-of-concept, pragmatic, multicentre, adaptive, open-label strategy trial embedded into routine clinical practice with stratified individual patient randomisation. The trial was co-designed with a patient and public involvement team. The intervention is a digital portal that supports shared decision-making on hypertension therapy class using clinical features plus metabolomic profiles determined with liquid chromatography-mass spectrometry.

Eligible patients are aged≥18 years with a systolic blood pressure≥140 mm Hg and a clinical indication for antihypertensive therapy. 400 participants will be randomised to the usual standard of care for treatment selection or to the intervention group. Remote follow-up will occur through a patient smartphone application and linked blood pressure monitor to assess the primary outcome of change in home systolic blood pressure during a 4-week period after medication changes. Secondary outcomes will include patient-reported adverse effects and quality of life, treatment withdrawal, healthcare utilisation and a health economic analysis. In the second phase of the trial, all participants will receive an updated version of the intervention regardless of the original randomised group.

**Ethics and dissemination:**

Ethical approval will be obtained for all sites. Approval in England: North West – Greater Manchester West Research Ethics Committee (REC) (25/NW/0296). Trial results will be disseminated via peer-reviewed publications and plain-language patient summaries.

**Trial registration number:**

NCT07294794 and ISRCTN29385951.

**STRENGTH AND LIMITATIONS OF THIS STUDY**

- The HYPERMARKER trial uses a multicentre randomised controlled design to evaluate a pharmacometabolomic-guided approach to aid antihypertensive drug class selection.
- HYPERMARKER was co-created with patient and public representatives, using digital technology to facilitate shared care and patient empowerment.
- This is a rapid sequence proof-of-concept trial designed to evaluate the potential for future pharmacometabolomic strategies to aid clinical decision-making; it will not assess longer-term outcomes.

## Introduction

 Hypertension affects over 30% of adults worldwide, with global prevalence having doubled between 1990 and 2019.^1 2^ Hypertension is a leading modifiable risk factor for cardiovascular, cerebrovascular and renal disease, leading to substantial morbidity and mortality.^3^ Despite being readily identifiable and treatable, it is often poorly managed. Globally, only 23% of women and 18% of men with hypertension achieved blood pressure levels within recommended target ranges in 2019.^1^ As a consequence, hypertension has a major economic impact, which could be substantially reduced through improving the use of existing drug therapy.^4^ Regardless of other patient comorbidities, reducing blood pressure has a significant patient benefit, including around a 10% lower risk of major cardiovascular events for every 5 mm Hg drop in systolic blood pressure (SBP).^5^

While the management of hypertension may be multifaceted and include lifestyle interventions, most patients require long-term drug therapy. Current European and American guidelines advocate the use of initial low-dose dual combination therapy, using either a thiazide or thiazide-like diuretic, calcium channel blocker, ACE inhibitor (ACEi) or angiotensin receptor blocker (ARB).^6 7^ However, limited data exist to guide clinicians in their choice for each individual patient. Where recommendations are made, this often relates to cardiovascular comorbidities where antihypertensives may have a beneficial effect outside of blood pressure lowering.^8^ One year after initiation, adherence to blood pressure medication is less than 50%,^9^ highlighting how adherence and side effect profiles are critical to overall management^10 11^ and the need to consider each patient individually.^12^

### Current clinical practice in hypertension management

**Figure 1:**
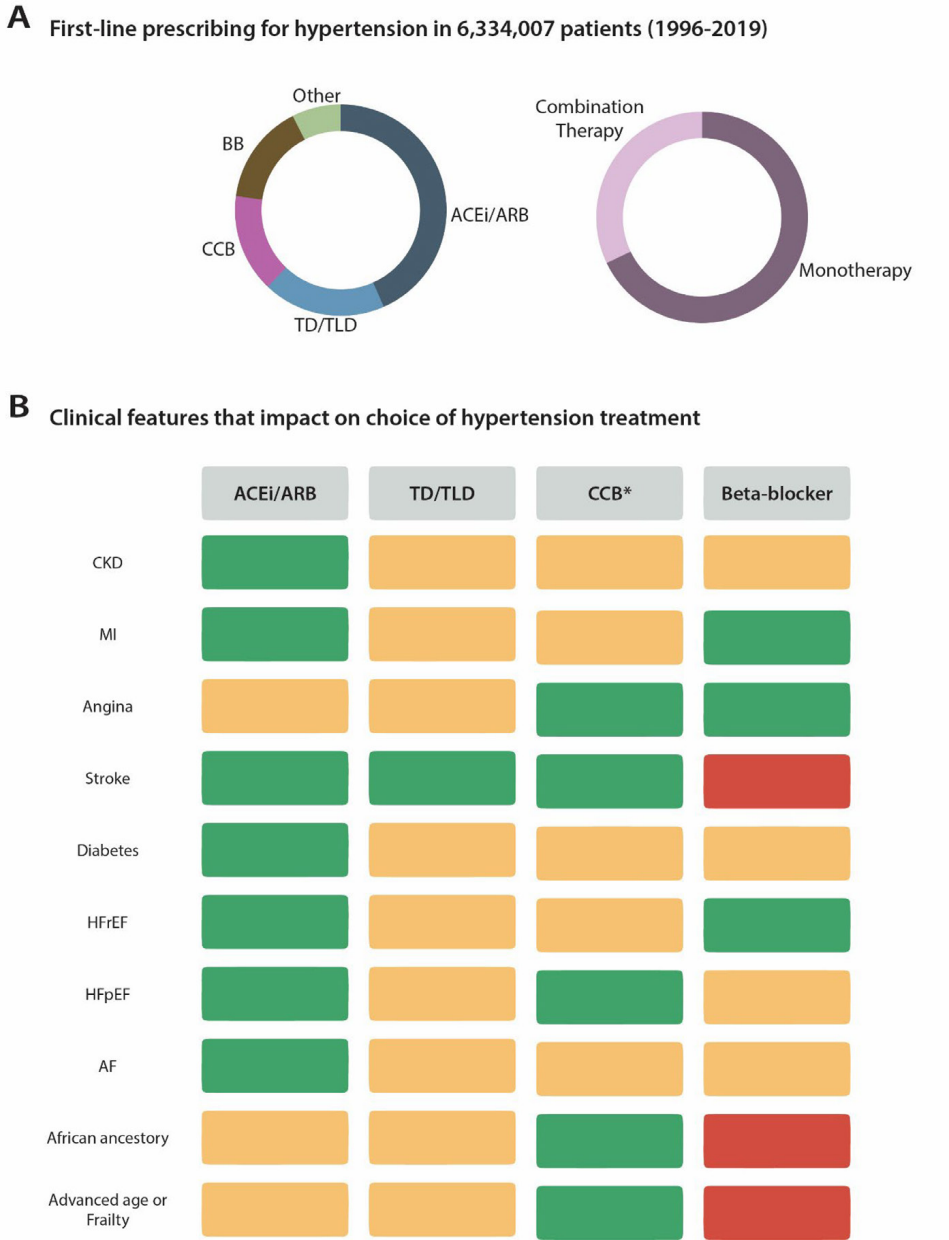
Choice of blood pressure-lowering treatment class in routine practice. (**A**) Pooled data from 6 334 007 patients enrolled between 1996 and 2019 across five studies using electronic healthcare records from the UK, the US, Germany, Italy and Canada.^13^,^17^ The drug classes used as initial therapy for study-defined hypertension were ACEi/ARB (43.3%), thiazide diuretics (18.8%), calcium channel blockers (15%), beta-blockers (15.4%) and other therapies (7.5%). Four of the five studies reported whether initial therapy was a single agent (67.9%) or combination therapy (32.1%) (n=4693,178 patients). (**B**) Clinical factors that may impact the most commonly prescribed drug classes for hypertension in clinical practice. Green, often used as first-line for hypertension management or another prognostic indication; amber, used in clinical practice but may not be first-line; red, typically avoided in clinical practice. * refers to dihydropyridine calcium channel blockers. ACEi, angiotensin-converting enzyme inhibitors; AF, atrial fibrillation; ARB, angiotensin II receptor blockers; BB, beta-blockers; CCB, calcium channel blocker; CKD, chronic kidney disease; HFrEF, heart failure with reduced ejection fraction; HFpEF, heart failure with preserved ejection fraction; MI, myocardial infarction; TD, thiazide diuretic; TLD, thiazide-like diuretic.

To gain real-world perspectives on hypertension management, we pooled data on the management of hypertension across five large studies using electronic healthcare records. In total, 6 334 007 patients with hypertension were included from the UK, the US, Germany, Italy and Canada, enrolled between 1996 and 2019.^13^,^17^ The drug classes used as initial therapy for study-defined hypertension were ACEi/ARB (43.3%), thiazide or thiazide-like diuretics (18.8%), calcium channel blockers (15%), beta-blockers (15.4%) and other therapies (7.5%) (figure 1A). Four of the five studies (n=4 693 178 patients) reported whether the initial therapy used a single agent or combination therapy, with combinations used in 32.1%. These figures represent a clear variation from current international guideline recommendations.

Reviewing consensus documents, guidelines and usual clinical practice, it is apparent that while there are several comorbid conditions that often dictate which drug class is used in a particular patient (figure 1B), the ability to personalise blood pressure treatment remains significantly limited in clinical practice. Moreover, these comorbid conditions are predominantly restricted to the context of secondary prevention and are often considered in isolation, not accounting for the substantial multimorbidity seen in those with hypertension.^18^

### Potential for personalised medicine

Personalising therapy can promote the selection of the most effective drug for an individual while avoiding therapies which are less effective for SBP reduction or associated with adverse effects. Substantial variability in the effectiveness of blood pressure treatment has been demonstrated in a systematic analysis of 135 population-based studies from 90 countries, underscoring the need for individualised approaches to hypertension management.^2^ While contributed by differences in awareness and access to healthcare, there are also inter-individual and inter-population differences in treatment response and adherence.^19^ Notably, differences in response between anti-hypertensive classes have been observed across 29 landmark hypertension trials.^20^ Randomised controlled trials (RCTs) confirm these major differences in blood pressure response and cardiovascular outcomes in underserved populations,^21^,^23^ with a complex treatment effect that is also impacted by the lived environment.^24^ In The Precision Hypertension Care repeated cross-over trial of single antihypertensive drugs, substantial differences in treatment response were demonstrated, with a modelled gain of 4.4 mm Hg for a personalised ‘best treatment’ approach versus a fixed choice.^25^ However, an evidence gap remains in guiding personalised therapy that frequently requires combination therapy^26^ in line with current hypertension guidelines.^6 7^ Metabolomics provides such an opportunity, quantifying thousands of metabolites that reflect an array of individual factors from genetic to environmental contributors that could be used to predict the response to treatment (pharmacometabolomics).^27 28^

The HYPERMARKER consortium was formed to address key evidence and implementation gaps—whether it is possible to personalise the choice of medication class for patients with hypertension and how to empower patients to take an active role in shared treatment decisions. To enable personalised selection of antihypertensive drug classes, machine learning approaches will isolate and combine conventional clinical features with metabolomic profiles generated via state-of-the-art high-resolution mass spectrometry.

### The HYPERMARKER trial

The HYPERMARKER trial is the first RCT to evaluate a pharmacometabolomic-guided approach to aid antihypertensive drug class selection.^29^ The trial is a proof-of-concept, pragmatic, adaptive, open-label strategy trial embedded in routine clinical practice and will use stratified individual patient randomisation. The primary objective of the trial is to determine the effect of a pharmacometabolomic-guided drug class approach on home SBP compared with standard of care (null hypothesis: no difference in home SBP). A further key objective is to develop, test and iterate a strategy of citizen engagement that can support personalised decision-making for hypertension treatment, informing the development of pharmacometabolomic-guided medical devices. The study is sponsored by the University of Birmingham and funded by EU Horizon and UK Research and Innovation.

## Methods and analysis

### Protocol

The full protocol for the HYPERMARKER trial is available as an online supplemental file 1.

### Patient and public involvement

The HYPERMARKER project is supported by a patient and public involvement (PPI) group. PPI representatives were recruited through the European Heart Network, Brussels and represent each proposed trial site country. The PPI group was involved in the study design and development of the trial protocol. Through focus groups, outcome measures were reviewed to ensure they represented the groups’ priorities and experience in prior treatment of hypertension. Trial procedures have been tested by the PPI team to ensure they are suitable and any burden is balanced and acceptable, including the home blood pressure device, mobile application and patient-reported outcome questionnaires. Participant recruitment material has been co-developed with the PPI team, with participant information videos featuring members of the PPI team. The PPI groups will work with researchers during the trial with representation on the trial Joint Oversight Committee.

### Enrolment

Four hundred eligible patients will be invited to participate in the study during routine secondary care encounters across multiple hospital sites in Europe. The inclusion criteria are patients aged over 18 years with an SBP≥140 mm Hg and a clinical indication for starting or adding antihypertensive therapy. Exclusion criteria are limited in order to maximise generalisability of findings (table 1). All participants will provide written informed consent on an approved form (online supplemental file 3).

**Table 1:** Inclusion and exclusion criteria

Inclusion criteria	Age 18 years or older. SBP≥140 mm Hg on any blood pressure recording method (office, home or ambulatory). Clinical indication for antihypertensive therapy.
Exclusion criteria	SBP≥180 mm Hg on any blood pressure recording method (office, home or ambulatory). Three or more current antihypertensive medications. Potential secondary cause of hypertension, including but not limited to renovascular hypertension, endocrine conditions, chronic kidney disease, coarctation of the aorta or medication-related causes. Planned intervention for hypertension, such as renal denervation. Severe kidney disease (eGFR<30 mL/min). Diagnosis of known heart failure with an LVEF<40%. Stroke or myocardial infarction within the last 6 months. Pregnancy, planning for pregnancy or breastfeeding. A participant whom the clinical investigator deems otherwise ineligible.

eGFR, estimated glomerular filtration rate; LVEF, left ventricular ejection fraction; SBP, systolic blood pressure.

### Intervention

The intervention (pharmacometabolomic approach) provides person-specific drug class decision support that clinicians may use when making their choice of blood pressure-lowering medication. This is generated using machine learning approaches that combine conventional clinical features with metabolomic profiles. In brief, leveraging data from multiple pan-European cohorts (online supplemental table 1), a rigorous pipeline was developed incorporating least absolute shrinkage and selection operator (LASSO) regression, XGBoost classification and Random Forest algorithms for model training to enable robust selection of drug-response features. Nested cross-validation was used for hyperparameter tuning and optimal model selection and stability-based ranking criteria for dominant feature selection. Full details on the pharmacometabolomic algorithm will be published at the end of phase 2 of the trial to avoid contamination across randomised groups. The standard of care involves usual clinical processes for choosing medication class and aligns with the 2024 European Society of Cardiology Guidelines for the Management of Elevated Blood Pressure and Hypertension.^6^

### Randomisation and allocation

**Figure 2:**
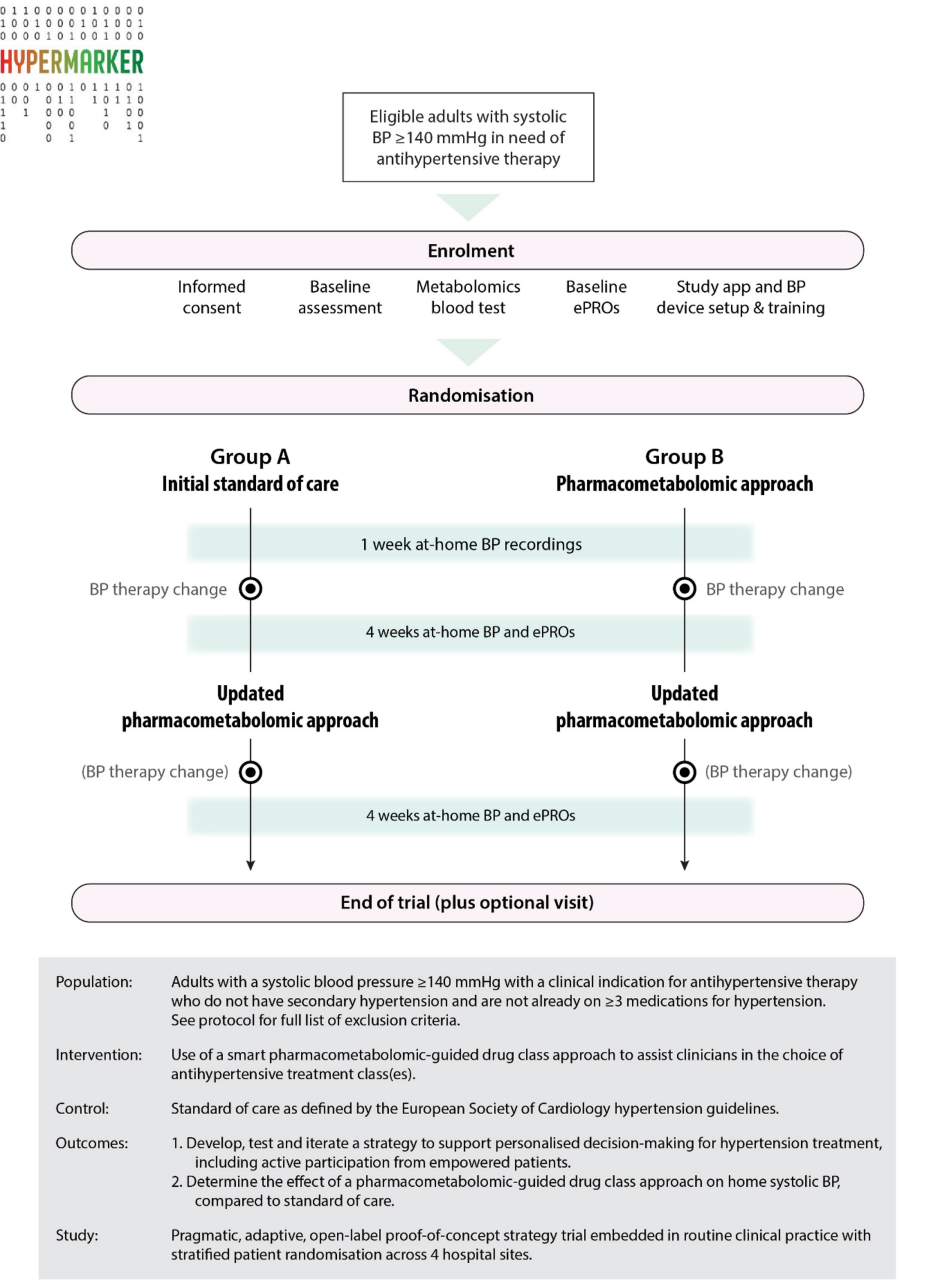
Schematic overview of the HYPERMARKER trial design. BP, blood pressure; ePROs, electronic patient-reported outcomes.

Following written consent, participants will be randomised in a 1:1 ratio to either the initial standard of care (Group A) or initial use of the pharmacometabolomic approach for treatment selection (Group B). Randomisation will be stratified by site (four sites), participant age (18–69 and ≥70 years) and baseline office SBP (140–159 mm Hg or ≥160 mm Hg). As an open-label trial, both the investigator and participant will be aware of the allocation after randomisation. The pharmacometabolomic approach will be updated as additional data are obtained during the trial. Participants will have their medication class re-reviewed, with the updated pharmacometabolomic output provided to investigators for all participants, regardless of original randomised group, to ensure equity (figure 2).

### Study procedures

Baseline assessments at enrolment include collection of participant demographic data, medical and medication history and patient-reported outcome questionnaires, including quality of life and food intake questionnaires and use of healthcare services in primary and secondary care. Individual metabolomic profiles are generated using a plasma sample taken at baseline and processed using a high-throughput liquid chromatography-mass spectrometry assay. Following enrolment, the trial will be conducted remotely with the provision of a home blood pressure device and smartphone application. Each participant will be asked to capture at least 1 week of home blood pressure measurements after randomisation and before changes to medications are made. A further 4 weeks of home blood pressure recordings will be requested after each medication change/review, with patient-reported quality of life, medication adherence, adverse events and healthcare consumption recorded at the end of each intervention phase via questionnaires completed using the participants' own electronic device (figure 2).

Home blood pressures will be measured using a semi-automatic, validated and Conformité Européenne (CE)-marked monitor (Rossmax Z5). Using a secure Bluetooth protocol, measurements are transferred to a mobile application (Viduet Health) on the participants' phones and then securely transferred to a joint patient, physician and researcher portal to inform medication review. Home blood pressure measurements will be taken for at least 3 days per monitoring week, with participants asked to record at least two measurements, taken in the morning and evening on each day.

Plasma will be extracted from non-fasted EDTA samples collected at enrolment and frozen to −80°C prior to shipment to the Biomedical Metabolomics Facility Leiden in the Netherlands. Analysis will be performed via reversed-phase liquid chromatography-mass spectrometry. Metabolites and matched internal standards will be detected in both positive and negative ion modes with a time-of-flight mass range from 60 to 800 Da after separate injections. Analyst Software (AB SCIEX, V.1.7.1) will be used for data acquisition. All targets are integrated in Sciex OS (AB SCIEX, V.4.0.0), with method blanks and quality control samples injected along the batch as a reference and to calculate quality performance characteristics. A workflow specific for the clinical trial setting will be used to perform batch correction. An anchor batch will be used to establish compound-specific linear regression models, which are then applied to back-calculate areas from internal standard-corrected ratios in subsequent trial batches after correcting for inter-batch differences using long-term quality control samples. The quantified metabolites of interest will be combined with conventional clinical variables within the machine learning-based tool to provide an estimated probability of beneficial treatment response according to hypertension drug class.

### Study outcomes

The primary outcome is change in home SBP comparing the intervention and standard of care groups. The major secondary outcomes are rate of SBP change, change in diastolic blood pressure, proportion of participants achieving guideline-defined target blood pressure, patient-reported adverse effects, quality of life, proportion of participants reporting treatment withdrawal and adherence. In addition, HYPERMARKER will demonstrate methods for direct patient involvement and empowerment, as well as healthcare pathways and health economic evaluation to underpin future implementation (online supplemental table 2) for full list of trial outcomes).

### Sample size

The sample size of 400 patients using home SBP provides 99% power to detect a 1.5 mm Hg difference in SBP between the pharmacometabolomic approach and standard of care groups. Sample size calculations are based on the expected minimum of 12 baseline SBP measurements and 48 follow-up measurements by the end of the first phase and assume a 2-sided alpha of 0.05, baseline SBP of 145 mm Hg (SD 20), with a correlation between SBP measurements of 0.7.^30^ Power is retained at >85% for a 2.5 mm Hg difference in home SBP even if only 3 baseline and 12 follow-up measurements are recorded (with correlation 0.5- and 2-sided alpha 0.05).

### Statistical analysis

A statistical analysis plan will be drafted and agreed upon prior to database unlock. The primary analysis will follow the intention-to-treat principle, analysing all participants in their originally assigned groups regardless of compliance with the allocated group and with no imputation for any missing data. Sensitivity analyses will include ‘as-treated’ and ‘per-protocol’ analyses. The primary outcome of change in home SBP using multiple repeated measurements will be analysed using generalised linear models using a random-effects estimator and exchangeable correlation matrix. These account for all available SBP readings, accommodate variability in measurement frequency and intervals between participants and account for correlation between repeated measures. Models will include adjustment for variables used in the randomised stratification (age and site), as well as gender, prior blood pressure treatment and relevant comorbidities. Secondary outcomes will be analysed using the same methods as described above (adjusted mean difference (for continuous outcomes), logistic or log-binomial regression (for categorical outcomes), χ^2^ or Poisson regression (for count data) and incidence rate ratio (for adverse events). A p value<0.05 will be considered statistically significant.

### Trial oversight, management and registration

Trial oversight, management and registration of the HYPERMARKER trial will be conducted in accordance with guidelines for Good Clinical Practice (GCP) and the Declaration of Helsinki. A joint oversight committee will comprise a Trial Steering Committee and an independent Data Monitoring Committee. Oversight will operate in accordance with trial-specific charters, based on the DAMOCLES recommendations.^31^ The trial is registered at ClinicalTrials.gov (NCT07294794) and ISRCTN (ISRCTN29385951). The trial protocol was developed in accordance with the Standard Protocol Items for Randomised Trials (SPIRIT) statement,^32^ the SPIRIT-PRO extension^33^ and the SPIRIT-Outcomes extension.^34^

### Ethics and dissemination

Ethical approval will be obtained for all trial sites. The North West – Greater Manchester West Research Ethics Committee (REC) (25/NW/0296) and the National Health Service Health Research Authority (IRAS project: 354889) have approved this study in England and Wales, UK. All findings of clinical relevance will be submitted to a suitable medical journal for publication after review by the oversight committees and the PPI team. The PPI team will provide a plain language summary of results that will be published alongside the scientific paper and sent to trial participants. Trial results will be reported in accordance with the CODE-EHR standards^35^ and CONSORT-AI reporting guidelines.^36^

## Discussion

Personalising antihypertensive therapy selection to reflect individual variability in treatment response may help to address a key area of need and reduce the substantial and preventable burden of hypertension.^25 37^ The HYPERMARKER trial seeks to address key evidence gaps on how personalised therapy could be implemented, how patients can be empowered to take an active role in shared treatment decisions and to determine whether individualised decisions on hypertension medication class are effective. HYPERMARKER will be the first time that person-specific drug class decisions using metabolomic profiles are tested within a RCT, using recent advances in machine learning.

**Figure 3:**
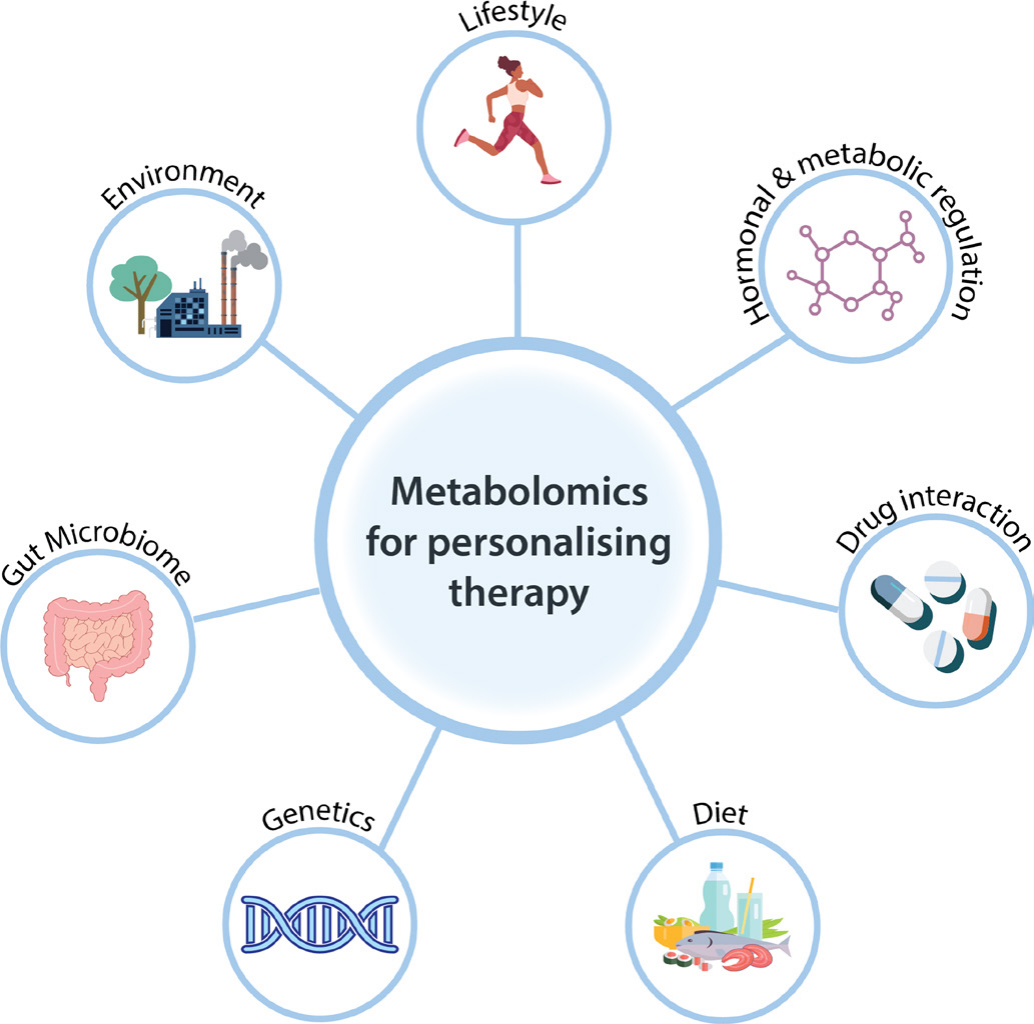
Span of individual patient factors related to metabolomics.

The pooled assessment of hypertension studies identifies a key need to individualise therapeutic approaches. Metabolomics offers a potential avenue to personalise antihypertensive therapy, with the composition and concentration of metabolites influenced by endogenous processes and their interactions with an individual’s lived environment that reflects the multifaceted aetiology of hypertension (figure 3). Metabolomics can facilitate differentiation of primary and endocrine hypertension, as well as identify potential markers of disease development and progression across different population groups.^38^,^45^ Potential metabolite biomarkers of blood pressure response to dietary and antihypertensive interventions have previously been demonstrated.^46^ N-methylglutamate, an amino acid linked to the intake of fruits, was associated with a significant reduction in SBP response after 4 weeks of a low-sodium diet compared with a high-sodium control diet (p=0.031).^47^ Arachidonoyl-carnitine was associated with a reduced SBP response to atenolol by 3.15 mm Hg (p=0.006).^48^ While the potential for pharmacometabolomics shows promise, most studies in this field only test the effect of monotherapies, which no longer aligns with current clinical guidelines. An evidence gap remains on how different dimensions of individual factors, including both clinical and metabolomic information, can be integrated to guide personalised treatment selection.

Machine learning can identify key variables from large, diverse biomedical and clinical datasets to generate highly accurate predictive models that can be validated across populations.^49 50^ It offers a potential pathway to personalised therapy and has already been applied to predict antihypertensive treatment response using standard clinical factors.^51^,^53^ However, large-scale studies that integrate metabolomic profiles into these models are lacking. Moreover, such models have not yet been tested in clinical trials, limiting their potential for clinical implementation.^54^

Digital tools for personalised therapeutics can significantly improve compliance and empower patients to take an active role in their healthcare decisions.^55 56^ A combined trial intervention including patient self-management and monitoring, with tailored titration, lifestyle advice and behavioural support, resulted in a mean difference in SBP of −3.4 mm Hg (95% CI −6.1 to −0.8 mm Hg) compared with usual care after 1 year (n=622).^57^ In an open‐label trial using daily home self‐monitoring with a linked smartphone app, personalised dosing of amlodipine achieved an SBP reduction of 11 mm Hg (95% CI 10 to 12, p<0.001, n=205) over 14 weeks, with high patient-reported adherence (94%).^58^ However, an individual patient data meta-analysis of randomised trials found that self-monitoring alone does not improve BP but requires a co-intervention.^59^

The HYPERMARKER trial, which includes PPI co-creation and digital tools that can link engaged patients with their healthcare provider, was designed to drive more efficient and cost-effective choices in blood pressure reduction. The pragmatic design uses out-of-office blood pressure assessment to enhance reliability and provide better correlation with end-organ damage and cardiovascular outcomes.^60^ The approach has a number of methodological limitations. As a rapid sequence proof-of-concept trial, it was not designed to assess cardiovascular outcomes. Instead, the 4-week follow-up after each intervention aligns with the expected SBP lowering effect from therapy.^61^ Adherence to therapy in routine practice is variable, and there is the opportunity for digital tools to enhance uptake of drug prescription.^62^ As part of the trial, we will assess adherence to the home SBP assessment, with similar trials demonstrating 80% adherence to weekly at-home blood pressure monitoring.^57^ HYPERMARKER adds automated SBP capture from home devices and uses participant reminders to optimise adherence across both arms to mitigate limitations.

In summary, the HYPERMARKER trial will evaluate personalised medication class selection for patients with hypertension using a pharmacometabolomic machine learning approach. The trial will determine whether empowered patients taking an active role in shared treatment decisions can result in more effective and efficient use of existing drug therapies, with fewer side effects and better adherence.

## Supplementary material

10.1136/bmjopen-2026-117869 online supplemental file 1

10.1136/bmjopen-2026-117869 online supplemental file 2

10.1136/bmjopen-2026-117869 online supplemental file 3
