# Supplementary material for: Personalised approach to hypertension treatment: protocol for the HYPERMARKER randomised controlled trial
Source: BMJ Open. 2026 Jul 16;16(7):e117869. doi: 10.1136/bmjopen-2026-117869 (PMC13384146; doi:10.1136/bmjopen-2026-117869)
Supplement: online supplemental file 3 [file bmjopen-16-7-s003.docx]

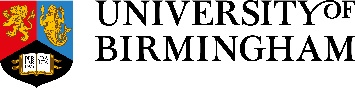
**
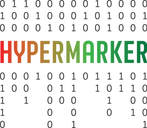
**

Add local

institution logo

**Personalised pharmacometabolomic-guided strategy trial to optimise treatment for hypertension (HYPERMARKER)**

**Centre Code: Participant Study ID Number:**

**CONSENT FORM**

|  | Please initial box |
| --- | --- |
| 1. I confirm that I have read the Participant Information Leaflet dated xxxxxx. (version XXX) for the above study. I have had the opportunity to consider the information, ask questions and have had these answered satisfactorily. |  |
| 1. I understand that my participation is voluntary and that I am free to withdraw at any time without giving any reason, without my medical care or legal rights being affected. I am aware that any information collected about me before I withdraw may still be used. |  |
| 1. I understand that I am required to download a smartphone application (app) and create a personal account with the app provider, Viduet Health, Netherlands for this study. I agree for my blood pressure data to be collected via the app, and understand it will be sent to the University of Birmingham during the study. |  |
| 1. I understand that my usual healthcare providers (such as my hospital or community practitioners) will be informed of my participation, and the research team may communicate with them during the study, including to share a summary of my blood pressure recordings and treatments. |  |
| 1. I understand that my blood pressure treatment may be changed during this trial, as outlined in the Participant Information Leaflet. I may be informed of these changes by electronic notification. My usual doctor will still manage my healthcare, and I will need to review my blood pressure treatment with them once I complete the study. |  |
| 1. I understand that this research study will collect and use my personal information as outlined in the Participant Information Leaflet, including from my available medical records. I give permission for the research team to access my medical records during the study. |  |
| 1. I understand that my privacy will be protected, and that my information may be processed in countries in the UK and European Union including with members of the HYPERMARKER research group during and after the trial as outlined in the participant information leaflet. Where I provide consent, this may also include such use in future studies. |  |
| 1. I agree to components of my blood sample(s) being sent, temporarily stored and analysed at Leiden University, Netherlands during the study. My sample will be given a code so I cannot be identified by Leiden University. |  |
| 1. I understand that relevant sections of my trial records and data collected during the study may be looked at by representatives of the Sponsor (University of Birmingham), regulatory authorities or the Hospital Trust, where it is relevant to my taking part in this research. I give permission for these individuals to have access to my records. |  |
| 1. I agree that the research team can contact me by telephone, text, email or via the study smartphone application during the study follow-up period. |  |
| 1. Where I choose to provide personal information, including my country of birth or ethnic origin, I am consenting for the processing of this data for the purposes of this research study. |  |
| 1. I understand that data collected about me will be used both during and after the study by the University of Birmingham for the ongoing development of the computer programmes (‘smart’ approach) being tested in this research study. |  |
| I agree to take part in the above study. |  |

|  | Please tick as appropriate | | Please initial box |
| --- | --- | --- | --- |
| 1. OPTIONAL: I agree to be interviewed and answer questions about my blood pressure care and attend a follow-up appointment if required (in-person, via online meeting or by telephone). I understand the interview may be audio recorded as outlined in the Participant Information Leaflet. | Yes  No | 🞎  🞎 |  |
| 1. OPTIONAL: I give my permission to be contacted in the future for other research studies. | Yes  No | 🞎  🞎 |  |

**______________________________________ _________________ _______________________________ Name of Participant Date Signature**

**If completed with the help of another individual:** I have provided an accurate representation of this information to the person mentioned above. They have stated they understand the information after an opportunity to have their questions answered.

**______________________________________ _________________ _______________________________ Name of individual providing assistance Date Signature**

**______________________________________ _________________ _______________________________ Name of Person seeking consent Date Signature**

*Original to be filed in the study file; one copy for the participant; one copy for the participant’s medical record.*
