# Supplementary material for: Personalised approach to hypertension treatment: protocol for the HYPERMARKER randomised controlled trial
Source: BMJ Open. 2026 Jul 16;16(7):e117869. doi: 10.1136/bmjopen-2026-117869 (PMC13384146; doi:10.1136/bmjopen-2026-117869)
Supplement: Supplementary file 4 [file bmjopen-2026-117869.reviewer_comments.pdf]

PEER REVIEW HISTORY

BMJ Open publishes all reviews undertaken for accepted manuscripts. Reviewers are asked to complete a checklist review form and are provided with free text boxes to elaborate on their assessment. These free text comments are reproduced below.

ARTICLE DETAILS

Title (Provisional)

Personalised approach to hypertension treatment: Protocol for the HYPERMARKER randomised controlled trial

Authors

Chapman, Matthew; Knikman, Jonathan E; Mobley, Alastair; Lagerwaard, Bart; Martinez-Garcia, Fernando; Zeitouny, Seraphine; Engler, Daniel; Schnabel, Renate B.; Spiering, Wilko; Bos, Iris D; Champsi, A; Carter, Alexander W; Hankemeier, Thomas; Grobbee, Diederick; Kotecha, Dipak; Consortium, Hypermarker

---

VERSION 1 - REVIEW

**Reviewer**	**1**
**Name**	**Sun, Lirong**
**Affiliation**	**Sichuan University, Cardiology Department**
**Date**	**11-Mar-2026**
**COI**	**None**

This protocol outlines a rigorously designed, innovative randomized controlled trial that addresses a pivotal gap in personalized hypertension management. The integration of metabolomic profiling with machine learning to inform antihypertensive medication selection is both timely and clinically impactful, aligning with the evolving paradigm of precision medicine. The pragmatic multicenter design, coupled with meaningful patient and public involvement throughout the study development, enhances the external validity and real-world applicability of the research.

The clearly delineated research objectives, robust randomization stratification strategy, and comprehensive suite of outcome measures—encompassing patient-reported outcomes, clinical endpoints, and health economic analysis—underscore the study's scientific rigor. To further strengthen methodological transparency, clarification of the core framework of the machine learning algorithm and specific quality control parameters for metabolomic analyses is recommended. Additionally, elaboration on strategies to ensure adherence among elderly participants and mitigate potential bias inherent to the open-label design would refine the protocol. Overall, this trial holds substantial promise for advancing

evidence-based hypertension care, and targeted minor revisions will optimize its methodological robustness and reproducibility.

**Reviewer**	**2**
**Name**	**Sever, Peter**
**Affiliation**	**Imperial College London, International Centre for Circulatory Health**
**Date**	**21-Apr-2026**
**COI**	**None**

This is a high risk but imaginative protocol for a trial in hypertensive patients designed to assess whether patient specific optimised treatment can be determined by pharmacometabolomic profiling of patients when compared with usual care. The outcome is determined by home measurements of blood pressure after 4 weeks of treatment where the intervention group have treatment choice determined by the pharmacometabolomic profile measured at baseline. The trial is powered to detect a systolic treatment difference between groups of 1.5 mmHg.

The pharmacometabolomic profile of the subjects will be determined not only by the multiplicity of pathophysiological variables that account for individual's blood pressure elevation, but also the extensive changes in profiling brought about by the 1-3 antihypertensive drugs that the patients have been taking prior to randomisation.

It is not clear from the protocol how information derived from the profiling will determine selection of individual drugs.

All subjects will be subject to treatment changes based on profiling at the second visit with further evaluation after 4 more weeks.

It is important that cost effectiveness studies are carried out because if only small differences are found, the substantial costs of intervention based on pharmacometabolomic profiling would surely preclude its use in day to day practice

I have no problem with the ethics and other aspects of the trial

Imaginative but high risk in my opinion (risk in the sense of obtaining a positive outcome)

VERSION 1 - AUTHOR RESPONSE

Reviewer 1

This protocol outlines a rigorously designed, innovative randomized controlled trial that addresses a pivotal gap in personalized hypertension management. The integration of metabolomic profiling with machine learning to inform antihypertensive medication

selection is both timely and clinically impactful, aligning with the evolving paradigm of precision medicine. The pragmatic multicenter design, coupled with meaningful patient and public involvement throughout the study development, enhances the external validity and real-world applicability of the research.

The clearly delineated research objectives, robust randomization stratification strategy, and comprehensive suite of outcome measures—encompassing patient-reported outcomes, clinical endpoints, and health economic analysis—underscore the study's scientific rigor. To further strengthen methodological transparency, clarification of the core framework of the machine learning algorithm and specific quality control parameters for metabolomic analyses is recommended. Additionally, elaboration on strategies to ensure adherence among elderly participants and mitigate potential bias inherent to the open-label design would refine the protocol. Overall, this trial holds substantial promise for advancing evidence-based hypertension care, and targeted minor revisions will optimize its methodological robustness and reproducibility.

Author Response:

We would like to thank the reviewer for their useful comments on our paper.

We have amended the methods and analysis section to provide an overview of the quality control parameter for metabolomic analyses and machine learning pipeline. It is planned that the full details of the pharmacometabolomic algorithm used throughout the trial will be published at the end of phase 2 of the trial through an additional methodological manuscript. This timing has been chosen to avoid contamination across randomized groups.

Adherence to home blood pressure measurements and therapy can be variable. Similar trials have demonstrated 80% adherence to weekly at-home blood pressure monitoring across a 3-week period.(McManus et al., 2021) The HYPERLINK cluster randomized controlled trial required a similar at-home measurement protocol to HYPERMARKER. In this study, participants took an average of 31.5 measurements per week. Measurement adherence was significantly higher in older age groups (56.3% in patients <49years, 69.8% in patients 49-65 years, and 79.5% in patients ≥ 65, $p<0.001$) (Kerby et al., 2012). To improve adherence to protocolized measurements and minimize bias, HYPERMARKER adds automated SBP capture from home devices. Automated participant reminders to optimise adherence across both arms will be used to mitigate limitations. In HYPERMARKER, participant reported adherence to therapy will be collected following each phase of the trial. We have updated the discussion section of the manuscript to account for the reviewer's comment.

Manuscript Changes:

Methods and analysis:

Page 10, Lines 194-204

The intervention (pharmacometabolomic approach) provides person-specific drug class decision support that clinicians may utilise when making their choice of blood pressure-lowering medication. This is generated using machine learning approaches that combine conventional clinical features with metabolomic profiles. In brief, leveraging data from multiple pan-European cohorts (Supplementary Table 1) a rigorous pipeline was developed incorporating LASSO regression, XGBoost classification and Random Forest algorithms for model training to enable robust selection of drug-response features. Nested cross-validation was used for hyperparameter tuning and optimal model selection, and stability-based ranking criteria for dominant feature selection. Full details on the pharmacometabolomic algorithm will be published at the end of phase 2 of the trial to avoid contamination across randomised groups.

Page 11-12 , Lines 238-250

Plasma will be extracted from non-fasted EDTA samples collected at enrolment and frozen to -80 degrees Celsius prior to shipment to the Biomedical Metabolomics Facility Leiden in the Netherlands. Analysis will be performed via reversed phase liquid chromatography-mass spectrometry. Metabolites and matched internal standards will be detected in both positive and negative ion mode with Time-of-Flight mass range from 60-800 Da after separate injections. Analyst Software (AB SCIEX, Version 1.7.1) will be used for data acquisition. All targets are integrated in Sciex OS (AB SCIEX, Version 4.0.0), with method blank and quality control samples injected along the batch as reference and to calculate quality performance characteristics. A workflow specific for the clinical trial setting will be used to perform batch correction. An anchor batch will be used to establish compound-specific linear regression models, which are then applied to back-calculate areas from internal standard corrected ratios in subsequent trial batches, after correcting for inter-batch differences using long-term quality control samples.

Discussion:

Page 17, Lines 362-376

Adherence to therapy in routine practice is variable, and there is the opportunity for digital tools to enhance uptake of drug prescription.[61] As part of the trial, we will assess adherence to the home SBP assessment, with similar trials demonstrating 80% adherence to weekly at-home blood pressure monitoring.[56] HYPERMARKER adds automated SBP capture from home devices and uses participant reminders to optimise adherence across both arms to mitigate limitations.

Reviewer 2

This is a high risk but imaginative protocol for a trial in hypertensive patients designed to assess whether patient specific optimised treatment can be determined by pharmacometabolomic profiling of patients when compared with usual care. The outcome is determined by home measurements of blood pressure after 4 weeks of treatment where the intervention group have treatment choice determined by the pharmacometabolomic profile measured at baseline. The trial is powered to detect a systolic treatment difference between groups of 1.5 mmHg. The pharmacometabolomic profile of the subjects will be determined not only by the multiplicity of pathophysiological variables that account for individual's blood pressure elevation, but also the extensive changes in profiling brought about by the 1-3 antihypertensive drugs that the patients have been taking prior to randomisation. It is not clear from the protocol how information derived from the profiling will determine selection of individual drugs. All subjects will be subject to treatment changes based on profiling at the second visit with further evaluation after 4 more weeks. It is important that cost effectiveness studies are carried out because if only small *differences are found, the substantial costs of intervention based on pharmacometabolomic profiling would surely preclude its use in day to day practice.*

I have no problem with the ethics and other aspects of the trial
Imaginative but high risk in my opinion (risk in the sense of obtaining a positive outcome)

Author Response:

We would like to thank the reviewer for their helpful comments on our paper.

We have amended the methods and analysis section to provide an overview of the machine learning pipeline. Full details of the pharmacometabolomic algorithm used during the study are planned to be published in a separate methodological paper following the completion of phase 2 of the study. This will outline how information derived from the profiling was used throughout the study to aid individualized drug class selection in the intervention arms.

The cost of pharmacometabolomic profiling is acknowledged and cost effectiveness studies will be carried out as part of this study. To help inform this analysis, participants will complete a healthcare utilization questionnaire, developed by the article's authors from the London School of Economics at baseline and following each intervention phase of the trial. The health economic outcomes are outlined within supplementary table 2.

Manuscript Changes:

Methods and analysis:

Page 10, Lines 194-204

The intervention (pharmacometabolomic approach) provides person-specific drug class decision support that clinicians may utilise when making their choice of blood pressure-lowering medication. This is generated using machine learning approaches that combine

conventional clinical features with metabolomic profiles. In brief, leveraging data from multiple pan-European cohorts (Supplementary Table 1) a rigorous pipeline was developed incorporating LASSO regression, XGBoost classification and Random Forest algorithms for model training to enable robust selection of drug-response features. Nested cross-validation was used for hyperparameter tuning and optimal model selection, and stability-based ranking criteria for dominant feature selection. Full details on the pharmacometabolomic algorithm will be published at the end of phase 2 of the trial to avoid contamination across randomised groups.

Supplementary materials; supplementary table 2.

A separate health economics analysis will be conducted comparing any pharmacometabolomic approach versus standard of care to determine the net monetary benefit, incremental cost-effectiveness ratios, the cost-effectiveness planes and the cost-effectiveness acceptability curves, discounted at recommended values.

References

Kerby, T. J., Asche, S. E., Maciosek, M. V., O'Connor, P. J., Sperl-Hillen, J. M., & Margolis, K. L. (2012). Adherence to blood pressure telemonitoring in a cluster-randomized clinical trial. *J Clin Hypertens (Greenwich)*, *14*(10), 668-674. https://doi.org/10.1111/j.1751-7176.2012.00685.x

McManus, R. J., Little, P., Stuart, B., Morton, K., Raftery, J., Kelly, J., Bradbury, K., Zhang, J., Zhu, S., Murray, E., May, C. R., Mair, F. S., Michie, S., Smith, P., Band, R., Ogburn, E., Allen, J., Rice, C., Nuttall, J.,…Yardley, L. (2021). Home and Online Management and Evaluation of Blood Pressure (HOME BP) using a digital intervention in poorly controlled hypertension: randomised controlled trial. *Bmj*, *372*, m4858. https://doi.org/10.1136/bmj.m4858
